# Application of autoantibody markers based on phage display immunoprecipitation sequencing technology in the diagnosis of periprosthetic joint infections

**DOI:** 10.3389/fcimb.2026.1824616

**Published:** 2026-07-14

**Authors:** GangJie Chang, YangCheng Ma, JianZhong Xu

**Affiliations:** The First Affiliated Hospital of Zhengzhou University, Zhengzhou University, Zhengzhou, China

**Keywords:** autoantibodies, diagnosis, phage display immunoprecipitation sequencing (PhIP-Seq), prosthesis-related infections, serologic tests

## Abstract

**Background:**

Accurate diagnosis of periprosthetic joint infection (PJI) is critical yet challenging. The role of autoantibodies in PJI remains largely unexplored. In this study, we employed phage display immunoprecipitation sequencing (PhIP-Seq) to profile PJI-associated autoantibodies and assess their diagnostic utility in PJI.

**Methods:**

We collected plasma samples from a cohort of 45 patients, including 25 with PJI, 9 with aseptic loosening, and 11 post-primary arthroplasty without complications. Initially, PhIP-Seq screening was performed on 10 samples (five PJI and five post-primary arthroplasty) to identify significantly differentially expressed autoantibodies. Subsequently, the immunoglobulin G (IgG) levels of these specific autoantibodies were quantified in the plasma of all participants via ELISA, and statistical analysis was performed to evaluate their diagnostic performance in PJI.

**Results:**

PhIP-Seq identified IFT80 and VPS54 as PJI-associated autoantigens. ELISA confirmed significantly elevated levels of corresponding IgG antibodies in PJI patients compared to those with aseptic loosening or post-primary arthroplasty (*p* < 0.01). Both autoantibodies demonstrated preliminary diagnostic performance: IFT80 achieved an area under the curve (AUC) of 0.797 (sensitivity = 56.0%, specificity = 95.0%), while VPS54 yielded an AUC of 0.776 (sensitivity = 72.0%, specificity = 75.0%). A combined diagnostic model integrating both biomarkers further enhanced the diagnostic performance (AUC = 0.810, sensitivity = 68.0%, specificity = 80.0%).

**Conclusion:**

This study establishes IFT80 and VPS54 as novel autoantibodies in PJI. Their corresponding autoantibodies exhibit robust diagnostic potential, offering support for the development of serological assays to improve PJI diagnosis.

## 1 Introduction

The growing aging population has led to a rising number of total joint arthroplasty (TJA) procedures for advanced osteoarthritis (Guan et al., 2019). While TJA effectively alleviates pain and restores function—thereby improving quality of life, particularly for elderly patients (Krenn et al., 2025), it carries the inherent risk of periprosthetic joint infection (PJI) (Luo et al., 2024). PJI remains a primary and severe complication, with an incidence of 1.0%–2.5% (Fontalis et al., 2021). Its treatment typically involves prolonged antibiotics and multiple revision surgeries, often resulting in significantly diminished quality of life and an increased risk of mortality for patients (Dimopoulou et al., 2023; Moldovan, 2024). Given the increasing volume of joint replacements, the clinical and economic burden imposed by PJI is expected to grow substantially (Kurtz et al., 2007).

The clinical presentation of PJI is often nonspecific, posing a challenge for conventional diagnostic methods (Hantouly et al., 2023). Over the past decades, major societies, including the Musculoskeletal Infection Society (MSIS), the American Academy of Orthopaedic Surgeons (AAOS), and the European Bone and Joint Infection Society (EBJIS), have established diagnostic criteria (Osmon et al., 2013; Parvizi and Gehrke, 2014; McNally et al., 2021). These criteria integrate findings from microbial culture, serum and synovial fluid biomarkers, histopathological evaluation, and imaging, among other tests (Ometti et al., 2022). Despite these advances, the diagnosis of PJI remains particularly difficult in cases of chronic or low-virulence infection (Chen et al., 2024). Consequently, improving diagnostic accuracy is critical for guiding effective treatment (Yoon et al., 2017).

Serological biomarkers are the most widely used diagnostic tools for PJI due to their accessibility (Akcaalan et al., 2022). In clinical practice, the most commonly employed markers include erythrocyte sedimentation rate (ESR), C-reactive protein (CRP), and D-dimer (Parvizi and Gehrke, 2014). However, their diagnostic utility is often hampered by a lack of specificity, as their levels can also be elevated in various non-infectious inflammatory states, such as systemic inflammation or post-surgical trauma (Sigmund et al., 2021).

Autoantibodies are immunoglobulins produced by B cells that target self-antigens, generally classified into natural and pathogenic types (Paek et al., 2012). While autoantibodies are classically associated with autoimmune diseases for diagnostic and therapeutic purposes, recent evidence strikingly implicates infection as a key environmental trigger for their production (Mu et al., 2024). This infection-induced autoimmunity can critically influence the disease severity and long-term prognosis. A major translational challenge is the precise identification of infection-triggered autoantibodies to distinguish them from other origins, such as underlying autoimmune conditions (Rackaityte et al., 2023).

Phage display immunoprecipitation sequencing (PhIP-Seq) is an emerging high-throughput technology for the profiling of antibody repertoires. It enables comprehensive analysis of an individual’s humoral immune response, thereby linking specific antibody signatures to various disease states and autoimmune processes (Huang et al., 2024). Current platforms, which require minimal serum, can screen antibodies against the entire human proteome. As such, this approach enables the unbiased identification of new autoantibodies across diverse pathological or experimental conditions (Fleischer et al., 2024). However, its application in orthopedics, particularly with regard to PJI, remains largely unexplored (Tiu et al., 2022).

The present study aimed to identify novel diagnostic autoantibodies in PJI through comprehensive serological profiling using high-throughput PhIP-Seq technology. We characterized the autoantibody signatures that were specifically elevated during PJI and investigated their roles within the host’s immune response to prosthetic infection. The successful identification and validation of these biomarkers may facilitate rapid and accurate differentiation between PJI and aseptic failure, both pre- and postoperatively, ultimately improving the clinical outcomes and alleviating the economic burden of revision surgery.

## 2 Materials and methods

The present study was conducted following a complete and standardized research workflow including participant enrollment, grouping arrangement, biomarker discovery, and clinical validation.

Firstly, all eligible subjects were screened strictly according to predefined inclusion and exclusion criteria. A total of 45 qualified patients were enrolled and allocated into three groups with clear clinical definitions: group A is the PJI group (*n* = 25), group B is the aseptic loosening revision group (*n* = 9), and group C is the primary arthroplasty group (*n* = 11). Each group was set for distinct control purposes to eliminate confounding factors from surgical trauma and non-infectious loosening.

Secondly, in the discovery stage, considering the high cost and complex operation of PhIP-Seq technology, 10 representative plasma samples (five from group A and five from group C) were selected for high-throughput autoantibody profiling, the aim of which was to screen out the differentially enriched candidate peptide biomarkers closely related to PJI.

Finally, in the validation stage, all plasma samples from the full 45-patient cohort were used for quantitative detection via ELISA. Differences in the expression of candidate autoantibodies among the three groups were statistically analyzed, and receiver operating characteristic (ROC) curve analysis was performed to comprehensively evaluate the diagnostic performance including the sensitivity, specificity, positive predictive value (PPV), and negative predictive value (NPV) of these novel biomarkers.

### 2.1 Study population

#### 2.1.1 Case inclusion and exclusion criteria

The inclusion criteria were as follows: 1) age ≥18 years, with the gender unrestricted; 2) history of hip or knee arthroplasty (including primary, aseptic revision, or septic revision procedures); 3) availability of complete clinical documentation; 4) provision of informed consent for voluntary participation and follow-up assessments; and 5) availability of sufficient data to classify patients according to the 2018 International Consensus Meeting criteria for PJI.

For patients in the aseptic loosening cohort, the diagnosis was based on: i) clinical presentation of mechanical (weight-bearing) pain (Mjöberg, 2021); ii) radiographic findings of a progressive periprosthetic radiolucency exceeding 2 mm (Mjöberg, 2021; Buijs et al., 2025); and iii) systematic exclusion of infectious etiology (Buijs et al., 2025).

The case exclusion criteria include: 1) presence of a concurrent autoimmune disease [e.g., rheumatoid arthritis (RA), systemic lupus erythematosus (SLE), or psoriatic arthritis (PsA)]; 2) active infection or open wounds at remote sites within the 3 months before surgery/enrollment; 3) a history of chemotherapy, radiotherapy, or bone marrow transplantation; 4) severe hepatic or renal dysfunction; and 5) unavailability of more than 30% of key medical records or research blood samples.

#### 2.1.2 Baseline characteristics

We enrolled patients who underwent revision TJA at the Department of Orthopedics, The First Affiliated Hospital of Zhengzhou University, from May 2023 to August 2025. Based on the inclusion and exclusion criteria, 45 patients were included and assigned into three groups: the PJI group (*n* = 25), the aseptic loosening group (*n* = 9), and the primary arthroplasty group (*n* = 11). For unified presentation throughout the manuscript, these three cohorts are uniformly abbreviated as follows: group A represents the PJI group consisting of patients receiving revision surgery due to confirmed PJI (among the enrolled PJI patients, cases with positive microbial culture results and confirmed culture-negative PJI cases were both included, which conformed to the actual clinical constituent characteristics of the PJI population); group B denotes the aseptic loosening group comprising patients undergoing revision surgery for mechanical loosening without infectious lesions, which served as the differential diagnosis control; and group C refers to the primary arthroplasty group including patients who underwent uncomplicated initial joint replacement without loosening or infection, acting as the baseline control to exclude the immune responses induced merely by surgical trauma.

Retrospective sample size estimation was performed before formal data analysis. Based on published relevant studies, we preset the diagnostic specificity of the detected autoantibodies at 95%, set the confidence level at 95%, and allowable error at 10%. The calculated minimum required number of PJI-positive samples was approximately 22 cases. In this study, we finally included 25 patients in the PJI group, which satisfied the basic sample demand for preliminary diagnostic efficacy evaluation. Restricted by the single-center source, strict diagnostic standards, and clinical admission conditions, the total enrolled sample size was limited, which has been acknowledged as a major limitation of this research.

This study was approved by the Institutional Review Board of The First Affiliated Hospital of Zhengzhou University (approval no. 2025-KY-1789). Written informed consent was obtained from all individual participants included in the study.

### 2.2 Plasma collection and processing

Peripheral venous blood was collected from patients after an overnight fast on the second hospital day. Blood was drawn into EDTA-coated tubes and promptly centrifuged at 3,000 × *g* for 5 min at 4°C. The resulting plasma supernatant was aliquoted into sterile, nuclease-free microcentrifuge tubes and stored at −80°C to prevent repeated freeze–thaw cycles until batch analysis.

### 2.3 PhIP-Seq technical workflow

Given the high cost and technical intensity of PhIP-Seq, a discovery-stage design was applied. We initially included 10 samples (five PJI and five primary arthroplasty controls) for unbiased screening to identify candidate autoantibodies, which were subsequently validated in the full cohort of 45 patients using ELISA. This two-stage strategy represents a standard approach in high-throughput biomarker research.

#### 2.3.1 Construction of the phage display library

Protein sequences were obtained from public databases, including UniProt and the National Center for Biotechnology Information (NCBI). The complete human proteome was downloaded from NCBI, and after deduplication, a set of 53,943 non-redundant proteins was obtained for library design. Each protein sequence was computationally cleaved into linear peptides of 56 amino acids in length. A tiling strategy with a 50% overlap (28-amino acid offset) between adjacent segments was employed to ensure comprehensive coverage of potential linear epitopes. This length was selected because 56-mer peptides provide optimal coverage of full-length linear B-cell epitopes, maintain appropriate structural flexibility, and enhance specific antibody binding. In contrast, shorter peptides often only cover fragmented epitopes and may reduce the sensitivity and accuracy of autoantibody detection. Peptides shorter than 56 amino acids at the C-terminus of proteins were forward-padded, and peptides representing proteins shorter than 56 amino acids overall were padded at the N-terminus using a flexible “GSGS” linker sequence.

The designed peptide sequences were converted to the corresponding DNA sequences using the R programming environment. Codon optimization was performed by prioritizing high-frequency *Escherichia coli* codons to enhance the expression efficiency, followed by further optimization to achieve a GC content within the 40%–60% range. All designed sequences were flanked by 16-bp universal extensions for subsequent PCR amplification and cloning. The final oligonucleotide pool was synthesized commercially via high-throughput DNA synthesis (Twist Bioscience Corporation, Carlsbad, CA, USA). The synthesized library underwent initial quality control by high-throughput sequencing to verify sequence integrity and representation.

The oligonucleotide library was dissolved and quantified. For construction, 2 ng of the library was used as a template for PCR amplification. The amplified product was digested and ligated into a linearized T7 select phage vector using a commercial packaging kit. The ligation reaction was incubated at 16°C overnight. After quality control via agarose gel electrophoresis, the ligation product was incubated at 22°C for 2 h for *in vitro* packaging. The reaction was terminated by adding 90 μl of Luria–Bertani (LB) medium. A 10-μl aliquot was used to assess the initial packaging efficiency via plaque assay. Upon qualification, large-scale plating and amplification were performed. The amplified phage particles were eluted, and impurities were removed by centrifugation. The final library titer [plaque-forming units (PFU) per milliliter) was determined to ensure a complexity sufficient for subsequent immunoscreening.

#### 2.3.2 Immunoprecipitation

Serum samples were centrifuged at 12,000 × *g* for 20 min at 4°C to remove any residual particulates. The supernatant was carefully transferred and diluted 1:1,000 in PBST (phosphate-buffered saline containing 0.1% Tween-20) to a final volume of 1 ml. The diluted serum was added to wells of a pre-blocked 96-well plate. For each plate, negative control (no serum), blank control (buffer only), and positive control (serum with known reactivity) wells were established. The diluted and pre-cleared phage display library was then added to each well, and the plate was incubated overnight at 4°C with gentle rotation to allow antibody–peptide binding.

Following incubation, 20 μl of pre-washed Protein G magnetic beads was added to each well to capture the antibody-bound phage complexes. The plate was incubated at 4°C for an additional 4 h with gentle mixing. Subsequently, the plate was centrifuged at 500 × *g* for 3 min and placed on a magnetic stand. The supernatant was carefully removed and the bead-bound complexes washed twice with 200 μl of TBS buffer containing 0.1% Igepal CA-630. After the final wash, the beads were resuspended in 40 μl of sterile nuclease-free water, transferred into a new 96-well PCR plate, and heated at 95°C for 10 min in a thermal cycler to elute the bound phage particles. The eluate containing the enriched phage DNA was collected by magnetic separation.

The eluted phage DNA served as the template for a two-step PCR to construct the sequencing library. The first-round PCR used universal primers specific to the flanking regions of the phage vector to amplify the peptide-encoding inserts. The second-round PCR employed primers containing unique sample barcodes (indexes) and Illumina (San Diego, CA, USA) sequencing adapters to enable multiplexed sequencing. The PCR products were evaluated using agarose gel electrophoresis for the correct fragment size. Products from one to three plates (pooled at 3 μl per well) were combined, and the target DNA fragment band was excised and purified from the gel. The concentration and the size distribution of the final pooled library were quantified (e.g., using a Bioanalyzer or Qubit) before sequencing on an Illumina platform.

#### 2.3.3 High-throughput sequencing and data analysis

The constructed sequencing libraries were subjected to paired-end sequencing (150-bp read length) on an Illumina platform, with a target depth of 4 million reads per sample. Raw sequencing reads were demultiplexed based on sample-specific barcodes. Paired-end reads were merged and aligned to the reference peptide library to generate the raw count of reads uniquely mapped to each peptide for each sample, which served as the fundamental quantitative metric.

To identify peptides specifically enriched by serum antibodies, a statistical model was applied. Firstly, a background Poisson distribution for each peptide was modeled using the sequencing data from the unselected input phage library (pre-IP control). For each serum sample, three key metrics were calculated for every peptide based on its read count relative to the mock immunoprecipitation (IP) control: the enrichment fold change (EF), the normalized read count (NR), and the statistical significance represented as −log10(*p*-value) (−lgpv). Peptides were considered significantly enriched (positive hits) in a given sample if they passed the combined thresholds for EF, NR, and −lgpv.

For comparative analysis across the PJI and control cohorts, single-sample EF values were calibrated: the EF values for peptides confirmed as enriched were retained, while the EF values for all non-enriched peptides were set to 1 to minimize background noise. The positive peptide data from all samples were then integrated using the calibrated EF values as the quantitative signal for downstream analysis. Differential analysis between the disease (i.e., PJI) and control (i.e., aseptic loosening and primary revision) cohorts was performed using non-parametric Wilcoxon rank-sum tests on the calibrated EF signals. In addition, the differential presence/absence of positive hits between cohorts was assessed using Fisher’s exact tests. Corresponding *p*-values were calculated and adjusted for multiple testing as appropriate.

### 2.4 ELISA validation experiment (using absorbance as a quantitative indicator of antibody levels)

Recombinant human proteins (IFT80 and VPS54) were diluted to a final concentration of 0.25 μg/ml in carbonate–bicarbonate coating buffer (pH 9.6). A volume of 50 μl of the antigen solution was coated onto each well of a 96-well microplate and incubated overnight at 4°C. The following day, the coating solution was discarded and the plate washed three times with PBS containing 0.05% Tween-20 (PBST). Nonspecific binding sites were blocked by adding 100 μl of 2% bovine serum albumin (BSA) solution per well, followed by incubation at 37°C for 2 h.

After blocking, the plate was washed three times with PBST. Plasma samples were diluted 1:100 in PBST containing 1% BSA. A 50-μl aliquot of each diluted plasma sample was added to the wells and incubated at 37°C for 1 h. The plate was then washed five times with PBST and blotted dry on absorbent paper. Subsequently, 50 μl of horseradish peroxidase (HRP)-conjugated goat anti-human immunoglobulin G (IgG) secondary antibody (diluted 1:10,000 in 1% BSA-PBST) was added to each well and incubated at 37°C for 1 h, followed by another five washes.

For colorimetric detection, 50 μl of 3,3,5,5'-tetramethylbenzidine (TMB) substrate solution was added to each well. The plate was incubated in the dark at room temperature for 15 min. The enzymatic reaction was stopped by adding 50 μl of 2 M sulfuric acid (H_2_SO_4_) per well. Immediately, the optical density (OD) was measured at both 450 nm (primary wavelength) and 620 nm (reference wavelength) using a microplate reader. The final absorbance value for each sample was calculated as the difference between the OD at 450 nm and the OD at 620 nm (OD _450 nm_ − OD_620 nm_) to correct for optical imperfections in the plate. All samples were assayed in duplicate or triplicate, and the mean corrected absorbance was used for subsequent statistical analysis.

The ELISA performed in this study is semi-quantitative, which used OD values to reflect relative autoantibody levels. Absolute quantification using a standard curve with purified IgG or recombinant antigen was not performed, which represents a methodological limitation.

To ensure reproducibility, a common reference plasma sample was tested in parallel on each plate to monitor and normalize potential inter-plate variations.

### 2.5 Statistical analysis

Demographic and clinical characteristics, including age, sex, weight, height, body mass index (BMI), and surgical type, were collected and analyzed. Data were analyzed using IBM SPSS Statistics (version 23.0; IBM Corp., Armonk, NY, USA). Continuous variables conforming to a normal distribution are presented as the mean ± standard deviation (SD). Skewed continuous variables are expressed as median with interquartile range (IQR). Categorical variables are reported as counts and percentages.

Univariate comparisons across the three groups were performed using one-way analysis of variance (ANOVA) for normally distributed continuous variables, the Kruskal–Wallis *H* test for non-normally distributed or ordinal variables, and the chi-square test (or Fisher’s exact test) for categorical variables. When a significant overall difference was detected (*p* < 0.05), *post-hoc* pairwise comparisons were conducted: the least significant difference (LSD) test was used following ANOVA, and Dunn’s test was applied after the Kruskal–Wallis *H* test.

Box plots were generated using GraphPad Prism (version 7.0; GraphPad Software Inc., San Diego, CA, USA). Diagnostic performance was evaluated through ROC curve analysis using MedCalc Statistical Software (version 19.0.4; MedCalc Software Ltd., Ostend, Belgium). The area under the curve (AUC), Youden’s index, sensitivity, and specificity were calculated. Differences between the AUCs were compared, with a two-sided *p* < 0.05 considered statistically significant.

## 3 Results

### 3.1 Demographic data

A total of 45 patients were enrolled and assigned into three groups: the PJI group (*n* = 25), the aseptic loosening revision group (*n* = 9), and the primary arthroplasty control group (*n* = 11) (**Table 1**). The baseline demographic and clinical characteristics were comparable across all groups, with no statistically significant differences observed in the gender distribution (*p* = 0.888), age (*p* = 0.621), BMI (*p* = 0.313), or the affected joint location (*p* = 0.888).

**Table 1:** Patient characteristics by study group.

	Group A (*n* = 25)	Group B (*n* = 9)	Group C (*n* = 11)	*χ*^2^/*F*	*P*-value
Gender				*χ*^2^ = 0.24	0.888
Men	12	4	6		
Women	13	5	5		
Age (years)	66.0 ± 10.8	61.9 ± 16.6	61.6 ± 7.5	*F* = 0.48	0.621
BMI	23.6 ± 4.12	22.16 ± 3.18	24.65 ± 2.36	*F* = 1.19	0.313
Site				*χ*^2^ = 0.24	0.888
Hip	13	4	6		
Knee	12	5	5		

Continuous variables with normal distribution are expressed as the mean ± standard deviation, while non-normally distributed variables are expressed as median (interquartile range). Categorical variables are expressed as case numbers. Group A represents the periprosthetic joint infection (PJI) group, group B the revision group, and group C the replacement group. BMI, body mass index.

### 3.2 High-throughput autoantibody profiling by PhIP-Seq

From the above cohort, five plasma samples were randomly selected from each of the PJI group and the primary arthroplasty group. High-throughput autoantibody profiling via PhIP-Seq was performed for initial biomarker screening.

Statistical analysis identified three peptides with significant differential enrichment between the PJI and control groups (Wilcoxon rank-sum test, *p* < 0.05). In addition, a total of 17 peptides met the exploratory threshold of Ratio_variation ≥60%, as listed in **Table 2**.

**Table 2:** List of target peptides.

Peptide_ID	GeneName	Wilcox_pvalue	%Case	%Control	Ratio_variation (%)
#537495	VPS54	0.009701	100.00	20.00	80.00
#541899	IFT80	0.02537	80.00	0.00	80.00
#201526	ABR	0.072006	60.00	0.00	60.00
#541534	ARHGAP20	0.072006	60.00	0.00	60.00
#620291	PDE4A	0.072006	60.00	0.00	60.00
#213042	GNL2	0.072006	60.00	0.00	60.00
#537494	VPS54	0.072006	60.00	0.00	60.00
#517457	ZC4H2	0.072006	60.00	0.00	60.00
#544112	SLC17A6	0.072006	60.00	0.00	60.00
#583864	RAD54L2	0.072006	0.00	60.00	60.00
#515185	LHX9	0.072006	0.00	60.00	60.00
#367746	AMER2	0.072006	0.00	60.00	60.00
#319323	TICRR	0.072006	0.00	60.00	60.00
#476468	TMEM121B	0.072006	0.00	60.00	60.00
#478524	SHANK3	0.072006	0.00	60.00	60.00
#583863	RAD54L2	0.072006	0.00	60.00	60.00
#382131	ARID1B	0.074639	20.00	80.00	60.00

Among these, peptides #541898 (IFT80), #541899 (IFT80), and #537495 (VPS54) showed statistically significant enrichment.

**Figure 1:**
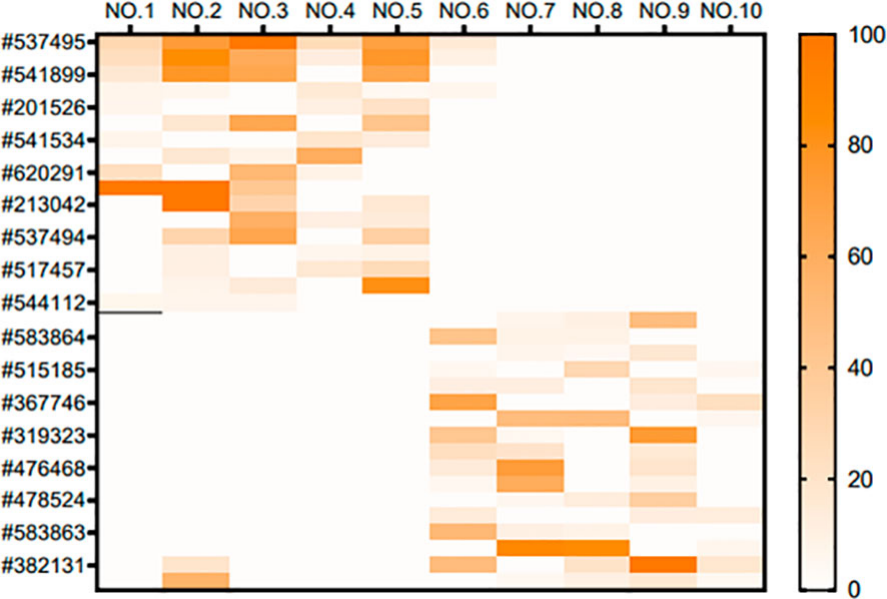
Heatmap of the differential peptides (Ratio_variation ≥ 60%). Each row represents the same peptide, while each column represents the same sample. Signal intensity is indicated by the corrected enrichment fold change (EF) value, with higher values denoting stronger signals, represented by deeper orange shades in the image. Numbers *1*–*5* denote the periprosthetic joint infection (PJI) group, and while *6*–*10* denote the primary arthroplasty group. The same applies below.

Specifically:

- Peptide #541898 (IFT80) was positive in 100% of the PJI samples and in 20% of the control samples (*p* = 0.0097).
- Peptide #541899 (IFT80) was positive in 80% of the PJI samples and in 0% of the control samples (*p* = 0.025).
- Peptide #537495 (VPS54) was positive in 100% of the PJI samples and in 20% of the control samples (*p* = 0.0097) (**Figure 1**).

These three peptides were regarded as core candidate biomarkers and were used for subsequent validation.

**Figure 2:**
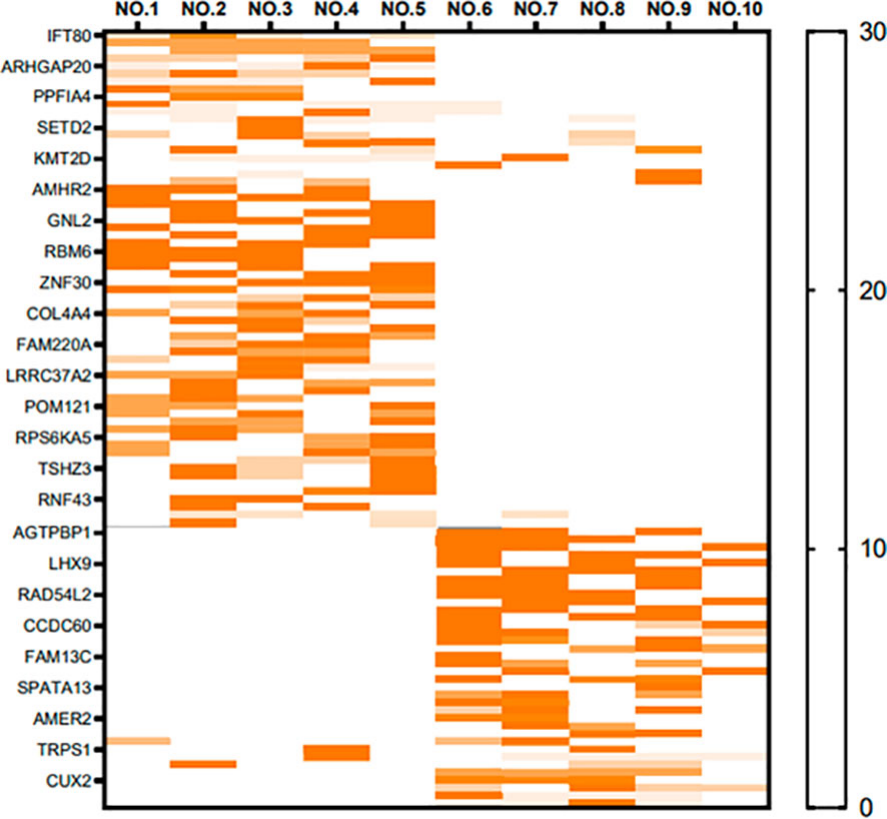
Differential gene heatmap. Each row represents the same gene, while each column represents the same sample. Signal intensity is expressed as normalized Hits percentage, with higher values indicating stronger signals, represented by deeper orange shades in the image.

At the gene level, IFT80 showed a positive rate of 100% in the PJI group *versus* 20% in the controls (*p* = 0.011751), further supporting its diagnostic potential (**Table 3**, **Figure 2**).

**Table 3:** List of target genes.

Gene name	Wilcox_pvalue	%Case	%Ctrl	Ratio_variation (%)
*IFT80*	0.011751	100.00	20.00	80
*VPS54*	0.096945	100.00	40.00	60
*ARHGAP20*	0.023151	80.00	0.00	80
*PPFIA4*	0.069642	80.00	20.00	60
*SETD2*	0.083265	80.00	20.00	60
*KMT2D*	0.248213	80.00	20.00	60
*AMHR2*	0.066798	60.00	0.00	60
*GNL2*	0.066798	60.00	0.00	60
*RBM6*	0.066798	60.00	0.00	60
*ZNF30*	0.066798	60.00	0.00	60
*COL4A4*	0.070701	60.00	0.00	60
*FAM220A*	0.070701	60.00	0.00	60
*LRRC37A2*	0.070701	60.00	0.00	60
*POM121*	0.070701	60.00	0.00	60
*RPS6KA5*	0.070701	60.00	0.00	60
*TSHZ3*	0.070701	60.00	0.00	60
*RNF43*	0.072006	60.00	0.00	60
*AGTPBP1*	0.066798	0.00	60.00	60
*LHX9*	0.066798	0.00	60.00	60
*RAD54L2*	0.066798	0.00	60.00	60
*CCDC60*	0.070701	0.00	60.00	60
*FAM13C*	0.070701	0.00	60.00	60
*SPATA13*	0.070701	0.00	60.00	60
*AMER2*	0.069642	20.00	80.00	60
*CUX2*	0.023151	0.00	80.00	80

### 3.3 Comparison of IFT80 and VPS54 among three groups

The plasma levels of the autoantibodies against IFT80 and VPS54, identified via PhIP-Seq, were quantified by ELISA in the PJI, aseptic revision, and primary arthroplasty groups. The results are presented as OD values in **Table 4**.

**Table 4:** Comparison of the VPS54 and IFT80 expression levels among the three patient groups.

Group	No. of cases	IFT80 OD value	VPS 54 OD value
PJI group	25	1.113 (0.689–1.560)	0.680 (0.469–1.297)
Refurbishment group	9	0.564 (0.342–0.854)	0.239 (0.134–0.554)
Total hip arthroplasty group	11	0.623 (0.368–0.913)	0.338 (0.216–0.648)

Optical density (OD) values: protein absorbance levels. Data are presented as the median (lower quartile–upper quartile).

PJI, periprosthetic joint infection.

ELISA quantification revealed significantly elevated plasma anti-VPS54 and anti-IFT80 antibody levels in the PJI group compared with both control groups (*p* < 0.01). The median OD values for both autoantibodies were robustly elevated in PJI patients. Furthermore, within the non-infected cohorts, the median OD values in the primary arthroplasty group were slightly higher than those in the aseptic loosening group.

**Figure 3:**
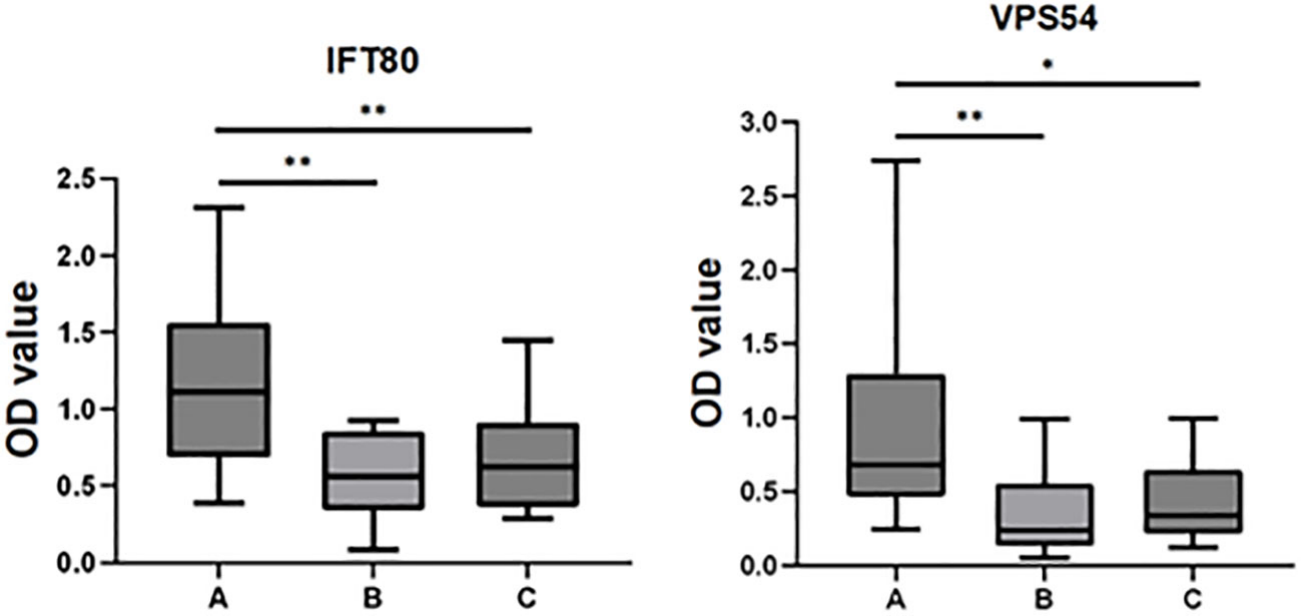
Group A represents PJI; Group B represents revision; Group C represents post-replacement; * p<0.05, ** p<0.01, *** p<0.001, **** p<0.0001.

The distribution of the anti-IFT80 and anti-VPS54 antibody levels (OD values) across the three patient groups is presented as box plots in **Figure 3**.

As the OD data for both autoantibodies violated the assumption of normality, non-parametric tests were therefore employed. Intergroup comparisons across the three cohorts were conducted using the Kruskal–Wallis *H* test, followed by Dunn’s *post-hoc* test for pairwise comparisons where significant differences were detected. The Kruskal–Wallis test revealed statistically significant differences in the plasma antibody levels among the PJI, aseptic loosening, and primary arthroplasty groups for both IFT80 (*H* = 11.51, *p* = 0.003) and VPS54 (*H* = 12.627, *p* = 0.002).

Dunn’s *post-hoc* tests detailed the pronounced elevation of both biomarkers in PJI. The median anti-IFT80 level was 1.113 (IQR = 0.659–1.560) in PJI, which is markedly higher than that in aseptic loosening (0.564; *p* = 0.008) and primary arthroplasty (0.623; *p* = 0.006) patients. Anti-VPS54 showed a parallel pattern, with a median of 0.680 (IQR = 0.469–1.297) in PJI *versus* 0.239 (*p* = 0.002) and 0.338 (*p* = 0.014) in the respective control groups. There were no statistically significant differences between the primary arthroplasty group and the aseptic loosening group for either autoantibody (*p* > 0.05). No trend toward difference is claimed because effect size data were not provided.

### 3.4 Value of IFT80 and VPS54 in the preoperative diagnosis of PJI

**Figure 4:**
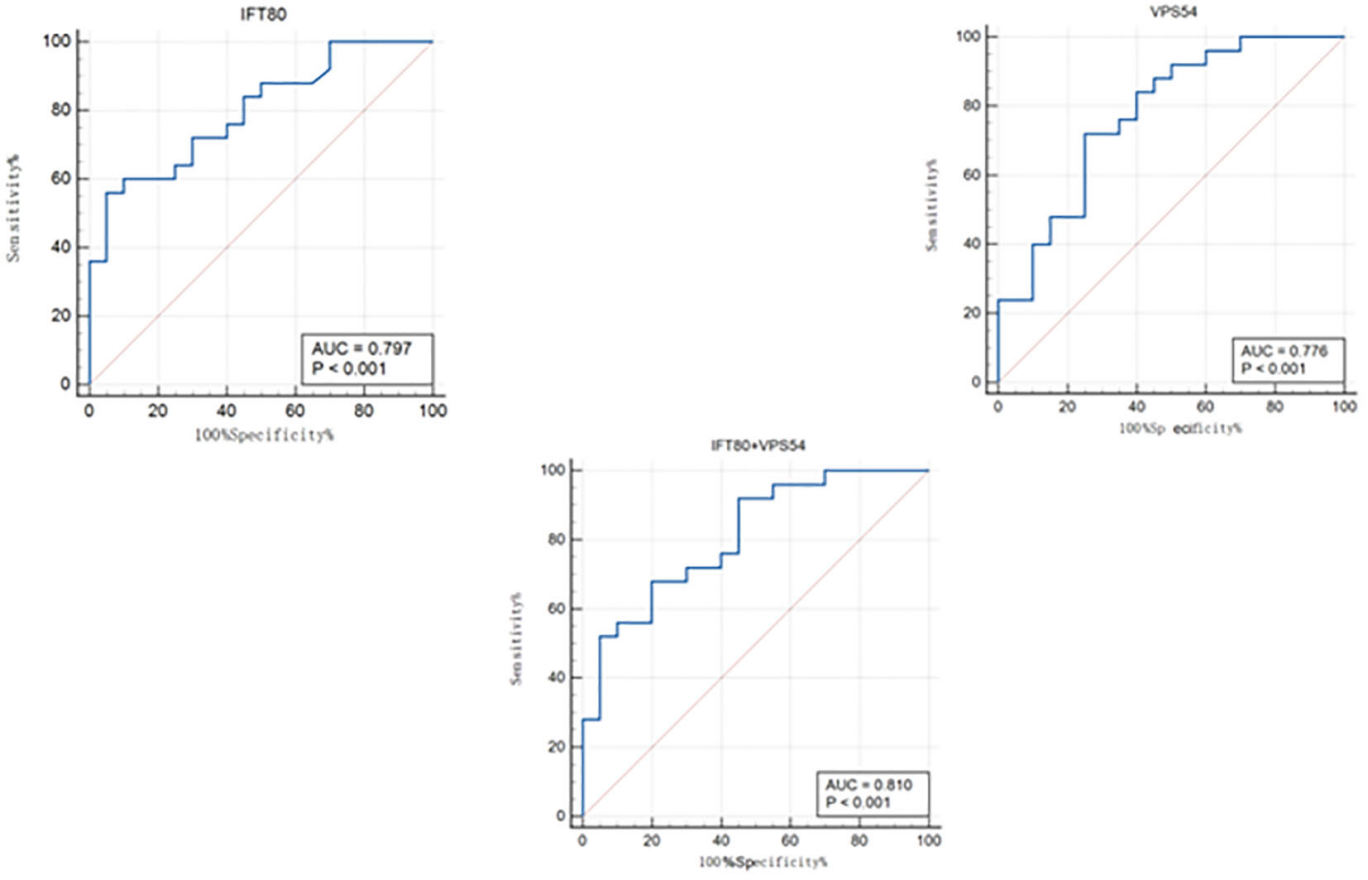
AUC denotes area under the curve; CI denotes confidence interval.

The diagnostic performance of the plasma autoantibodies was evaluated using ROC curve analysis. Both anti-IFT80 and anti-VPS54 antibodies demonstrated significant diagnostic value, with AUCs of 0.797 and 0.776, respectively (both *p* < 0.001 *versus* an AUC of 0.5) (**Figure 4**). At their optimal cutoffs, anti-IFT80 exhibited high specificity (95.0%), but moderate sensitivity (56.0%), whereas anti-VPS54 showed a more balanced profile (sensitivity = 72.0%, specificity = 76.0%). Notably, a combined diagnostic model integrating both markers achieved the highest overall efficacy, with an AUC of 0.810, a sensitivity of 68.0%, and a specificity of 80.0% (**Table 5**).

**Table 5:** Comparison of the diagnostic value of IFT80 and VPS54 for periprosthetic joint infection (PJI).

Marker	Cutoff value	AUC (95%CI)	Sensitivity (95%CI)	Specificity (95%CI)	PPV (%)	NPV (%)
IFT80	0.992	0.797 (0.651–0.902)	56 (34.9%–75.6%)	95 (75.1%–99.9%)	93.3	63.9
VPS54	0.468	0.776 (0.627–0.887)	72 (50.6%–87.9%)	75 (50.9%–91.3%)	75.0	72.0
IFT80+VPS54	0.528	0.810 (0.665–0.911)	68 (46.5%–85.1%)	80 (56.3%–94.3%)	81.0	66.7

UC, area under the curve; PPV, positive predictive value; NPV, negative predictive value.

## 4 Discussion

PJI is a devastating complication following TJA. While various biomarkers are currently in clinical use, their diagnostic accuracy remains suboptimal, particularly for chronic low-virulence infections (Fontalis et al., 2021; Dimopoulou et al., 2023; Luo et al., 2024; Krenn et al., 2025). As clinical management is contingent upon a precise diagnosis, refining the diagnostic strategies for PJI remains imperative. Through a systematic serological screening approach, the present study identified IFT80 and VPS54 as promising novel diagnostic biomarkers in PJI.

In contrast to traditional methods such as ELISA or Western blotting (WB), which are limited to the detection of antibodies against predefined antigens, PhIP-Seq employs serum antibodies as molecular probes to interrogate a library encompassing fragments of the human proteome (Huang et al., 2024). This approach identifies candidates such as IFT80 and VPS54 based on their interaction with patient antibodies, thereby establishing a functional link to the active humoral immune response in PJI. Consequently, PhIP-Seq findings are more likely to reveal pathologically relevant targets rather than non-specifically elevated proteins. A key translational advantage is the use of short peptide sequences (typically 12–20 amino acids) displayed on phage surfaces (Mohan et al., 2018). The enrichment of a specific phage clone not only reveals the target protein but also maps the exact linear epitope, providing the essential structural information required to engineer highly specific diagnostic assays and streamline clinical biomarker development (Andreu-Sánchez et al., 2023).

The present study identified autoantibodies against IFT80 and VPS54 as novel serological markers associated with PJI. While their roles in PJI are largely unexplored, IFT80 is a well-established regulator of bone metabolism (Yuan et al., 2019). Intraflagellar transport (IFT) proteins, including IFT80, are essential for osteoblast function, chondrocyte-mediated bone maintenance, cell polarity, and T-cell development. Primary cilia, whose assembly is dependent on IFT80, act as vital cellular mechano- and chemosensors (Lim et al., 2020). In osteoblasts, IFT80 regulates the Hedgehog/Gli signaling pathway via primary cilia to stimulate osteogenic differentiation (Chinipardaz et al., 2022), whereas its deficiency leads to shortened or absent cilia, blocking Hedgehog signaling and impairing osteoblast differentiation (Rix et al., 2011). Furthermore, IFT80 modulates TRPA1 expression and its mediated calcium influx, subsequently inhibiting the AKT and ERK pathways, thereby compromising mechanosensitive osteoblast differentiation (Wang et al., 2025). IFT80 also binds to Cbl-b to promote the ubiquitination and degradation of TRAF6, subsequently suppressing the RANKL signaling pathway and limiting osteoclastogenesis (Deepak et al., 2022). A hallmark of PJI is persistent bacterial infection and a robust inflammatory response, characterized by elevated cytokines that enhance osteoclast activity (Ren et al., 2025). We hypothesize that, in this inflammatory milieu, autoantibodies targeting IFT80 may arise as a compensatory immune response. This is a hypothesis-generating interpretation rather than a confirmed conclusion. By potentially modulating the Cbl-b-mediated TRAF6 degradation pathway, these antibodies might exert a protective function, counteracting the infection-driven bone destruction by promoting osteoblast activity and restraining osteoclastogenesis (Rix et al., 2011; Deepak et al., 2022; Wang et al., 2025).

The Golgi apparatus orchestrates cellular secretory and endocytic trafficking (Park et al., 2021). In this context, the Golgi-associated retrograde protein (GARP) complex—particularly its essential subunit VPS54—plays a key role in the maintenance of endosomal–lysosomal trafficking and organelle homeostasis (Oka, 2005). Our finding of elevated anti-VPS54 autoantibodies in PJI patients suggests a pronounced humoral response to this protein, which may reflect an underlying cellular stress (Dimopoulou et al., 2023). We hypothesize that the persistent inflammatory and reparative demands within the infected bone tissue disrupt endosomal trafficking, potentially upregulating VPS54 expression or altering its immunogenicity. Given the role of VPS54 in lysosomal maturation and its possible involvement in antigen presentation (Pérez-Victoria and Bonifacino, 2009; Patel et al., 2020), its targeting by autoantibodies could further perturb intracellular transport, cytokine secretion, and immune modulation—processes central to the pathology of PJI. Consequently, the ensuing humoral immune response against VPS54 might be a biomarker of this dysregulated cellular transport and secretory activity central to PJI pathology.

During the screening process, it was observed that both IFT80 and VPS54 exhibited significant differences at the peptide level, whereas only IFT80 showed a significant difference at the gene level. This phenomenon may be attributed to the regulation of VPS54 protein abundance or immunogenicity through post-translational mechanisms—such as modification, localization, or altered stability—which could enable it to elicit a strong autoantibody response even in the absence of significant changes at the transcriptional level.

The autoantibodies against IFT80 and VPS54 emerge as promising novel diagnostic markers for PJI, with clear advantages over conventional markers including CRP, ESR, and D-dimer. Conventional markers such as ESR and CRP suffer from suboptimal accuracy (Pérez-Prieto et al., 2017). ESR shows a sensitivity of 56% and a specificity of 75%, while CRP exhibits 74% sensitivity and 65% specificity (Wu et al., 2022; Xu et al., 2022; Wixted et al., 2023). Importantly, these markers are prone to false negatives in low-virulence infections and false-positive elevations in non-infectious inflammatory conditions (Akcaalan et al., 2022). In contrast, IFT80 demonstrated superior specificity (95%), and VPS54 offered better sensitivity (72%). A diagnostic model combining both markers balanced these attributes, achieving a sensitivity of 68% and a specificity of 80%. This performance surpasses that of traditional markers and underscores a viable strategy for the development of more accurate PJI diagnostics.

Clinically, a considerable proportion of PJIs are defined as culture-negative PJI, which is mainly caused by preoperative empirical antibiotic administration, low-virulence pathogenic infection, insufficient sampling volume, and slow-growing microbial characteristics (Lin et al., 2025). Traditional microbial culture is prone to false-negative results in such patients, leading to delayed diagnosis and improper clinical intervention.

Distinct from pathogen detection indexes, the anti-IFT80 and anti-VPS54 autoantibodies screened and verified in this study reflect sustained systemic humoral immune activation induced by long-term implant-associated infection. This host-derived immune signature is not easily inhibited by short-term antibiotic therapy and is independent of microbial culture outcomes (Park et al., 2023). Therefore, these novel serological biomarkers hold great potential to serve as effective auxiliary indicators for the diagnosis of culture-negative PJI, making up for the deficiency of traditional microbial culture methods and expanding the clinical applicable scope of PJI serological diagnosis. Further stratified analysis with an enlarged sample size will be carried out in follow-up studies to verify the actual diagnostic efficiency in a culture-negative PJI subgroup.

In summary, this study identified IFT80 and VPS54 as novel autoantibody biomarkers for PJI. The high specificity of IFT80 and the balanced performance of the combined panel provide a promising alternative to conventional serological tests. These biomarkers may help improve the accuracy and objectivity of PJI diagnosis, particularly in clinically challenging scenarios.

This study has several limitations that should be considered: 1) The limited sample size may have affected the robustness of the diagnostic performance estimates. Future validation in a large, multicenter cohort is required. 2) The ELISA used in this study was semi-quantitative without absolute quantification, which may have limited comparability between different batches. 3) The absence of control groups with other inflammatory joint diseases (e.g., RA) precludes definitive conclusions regarding the specificity of these markers for PJI *versus* general joint inflammation. 4) The specific pathogenic role, if any, of the anti-IFT80 and anti-VPS54 autoantibodies in PJI remains unexplored.

## 5 Conclusion

This study establishes IFT80 and VPS54 as novel serological biomarkers with diagnostic potential for PJI. Their differential expression, confirmed via PhIP-Seq and ELISA across distinct patient cohorts, underscores their translational promise. Prospective validation in large, multicenter studies is warranted to advance these findings toward clinical application.

## Data Availability

The raw data supporting the conclusions of this article will be made available by the authors, without undue reservation.
